# Finger Vein Identification Based on Large Kernel Convolution and Attention Mechanism

**DOI:** 10.3390/s24041132

**Published:** 2024-02-09

**Authors:** Meihui Li, Yufei Gong, Zhaohui Zheng

**Affiliations:** 1 School of Computer Science and Technology, Soochow University, Suzhou 215006, China; 20214227038@stu.suda.edu.cn; 2 Jiangsu Engineering Laboratory of Cyberspace Security, Suzhou 215006, China; 3 School of Software Engineering, Xi’an Jiaotong University, Xi’an 710049, China; gyf222@stu.xjtu.edu.cn

**Keywords:** finger vein identification, CNN, large kernel, attention mechanism, dual-channel

## Abstract

FV (finger vein) identification is a biometric identification technology that extracts the features of FV images for identity authentication. To address the limitations of CNN-based FV identification, particularly the challenge of small receptive fields and difficulty in capturing long-range dependencies, an FV identification method named Let-Net (large kernel and attention mechanism network) was introduced, which combines local and global information. Firstly, Let-Net employs large kernels to capture a broader spectrum of spatial contextual information, utilizing deep convolution in conjunction with residual connections to curtail the volume of model parameters. Subsequently, an integrated attention mechanism is applied to augment information flow within the channel and spatial dimensions, effectively modeling global information for the extraction of crucial FV features. The experimental results on nine public datasets show that Let-Net has excellent identification performance, and the EER and accuracy rate on the FV_USM dataset can reach 0.04% and 99.77%. The parameter number and FLOPs of Let-Net are only 0.89M and 0.25G, which means that the time cost of training and reasoning of the model is low, and it is easier to deploy and integrate into various applications.

## 1. Introduction

In recent years, noteworthy strides have been made in the realm of artificial intelligence, yielding substantial scientific research outcomes. Consequently, the field of biometric technology has entered a pivotal phase of advancement. However, the exposure of irregularities in the acquisition of facial identification technology data has redirected the focus of major research groups toward the exploration of more secure identification methodologies. One such method that has garnered considerable attention is FV identification, owing to its heightened security attributes. The intricate distribution of human finger veins beneath the skin, coupled with the absorption of near-infrared light by hemoglobin to generate vein images, distinguishes this modality. This distinctiveness, not easily replicated in everyday contexts, positions FV identification as a promising alternative. In comparison to prevalent technologies such as face and fingerprint identification, FV identification possesses four fundamental advantages: (1) The reliance on vein characteristics formed by human blood flow establishes it as a bona fide living body identification technology. (2) The in-body nature of FV features, embedded beneath the skin, confers inherent resistance to forgery. (3) It does not change with age, which means that enrollment can be used throughout a lifetime after adulthood [1]. (4) The identification process remains impervious to surface environmental factors, ensuring heightened security and significantly augmenting the identification pass rate.

The existing FV identification algorithms can be broadly classified into two categories: those relying on traditional handcrafted features [2,3,4], where researchers manually select features to effectively represent FV characteristics, and those employing deep learning for FV identification [5,6,7]. Traditional FV identification algorithms typically involve manual feature extraction, capturing both global and local features such as vein pattern, texture, and minutiae points. However, this approach exhibits several drawbacks, including a high requirement for image quality, dependency on manual preprocessing, insufficient algorithmic robustness, and the instability of extracted features, often leading to pseudo veins. To address these challenges, an increasing number of scholars are incorporating neural network technologies into FV identification methodologies. Convolutional neural networks (CNNs) have been widely applied in FV identification tasks and have demonstrated outstanding recognition performance [8,9,10]. This is attributed to their exceptional ability to extract and encode image features. Stacking convolutional layers and pooling layers, CNNs autonomously learn and capture local features in images, including vein textures, branch structures, and the spatial arrangement of local features, enabling a high-level abstraction and representation of FV characteristics. In traditional CNN structures, the operations of each convolutional kernel exhibit locality, meaning each kernel can only perceive the input region within its window size, known as the receptive field. Consequently, as the network depth increases, although higher-level features encompass larger global context information, the association of mid- to long-range features in lower layers may become blurred due to this locality, hindering the integration and recognition of global features. Additionally, the literature [11,12] indicates that features in FV images are not solely distributed in the vein regions but also exist in non-vein areas, referred to as soft biometrics, contributing to FV identification. Therefore, the limitation of the CNN receptive field poses a significant obstacle in extracting these comprehensive features. Furthermore, compared to other large-scale image recognition tasks, FV datasets, due to the challenges in their collection and ethical-legal constraints, often exhibit rich category diversity, but the sample size per category is considerably limited. This characteristic presents a severe challenge to the training and generalization capabilities of CNNs.

Hence, to address the prevalent issue of limited receptive fields in most CNN-based approaches, as well as the challenges associated with effectively capturing mid- to long-range dependencies within images and the scarcity of training samples during the model training phase, in this work, we introduce a novel model, named Let-Net, which strategically amalgamates large kernels and attention mechanisms. By integrating large kernels to expand the receptive field and incorporating an attention mechanism for optimized feature selection, Let-Net achieves a balanced optimization between recognition accuracy and computational cost. Experimental results demonstrate the outstanding performance of Let-Net across multiple datasets, while concurrently maintaining structural simplicity and computational efficiency. The key contributions of this study are outlined as follows:

(1) Pioneering the incorporation of a large kernel solution into the FV identification task, we introduce a large kernel structure named LK Block, featuring taper connection and hybrid depthwise convolution. This innovative architecture adeptly captures the comprehensive distribution and global features of finger veins via expansive convolution operations, consequently enhancing the speed and quality of feature extraction.
(2) The introduction of a module that integrates attention mechanisms and residual connections enhances information flows in channels and spaces by emulating visual attention mechanisms. This effectively mitigates limitations associated with convolutional induction bias and improves the discriminative capacity and robustness of features.
(3) By leveraging a dual-channel architecture, Let-Net effectively expands the dataset, seamlessly integrating feature comparison and extraction without explicit feature extraction steps. This innovative approach yields excellent identification results without the need to extract specific areas of interest.
(4) Experimental evaluations conducted across nine public datasets underscore Let-Net’s considerable advantages in the domain of FV identification. Notably, on the VERA dataset [13], characterized by its low quality, Let-Net outperforms current state-of-the-art methods by a significant margin.

## 2. Related Works

### 2.1. FV Identification Method Based on Deep Learning

Presently, advancements in deep learning technology have significantly propelled the field of facial verification (FV) identification. Radzi et al. [5] pioneered the application of CNNs to FV identification; however, their experiments, conducted on an internal dataset, lacked a comprehensive evaluation of generalization performance. In a corresponding way, Das et al. [8] introduced a CNN-based FV identification system, assessing its efficacy across four public datasets and achieving an accuracy of approximately 95%, though acknowledging the potential for improvement. In a different context, Yang et al. [9] presented FVRAS-net, an embedded FV identification system known for its lightweight and fast-forward propagation calculations. Nonetheless, its identification accuracy remains a focal point for enhancement, particularly highlighted by a 5.61% misidentification rate in the SCUT_RIFV [14] dataset. In pursuit of heightened accuracy, Shaheed et al. [15] devised an FV identification method based on the Xception model, integrating depthwise separable convolution and residual connections to interlink feature information between layers. Despite the method’s intricate network structure, it incurs higher computational costs. The recent state-of-the-art (SOTA) approach FVFSNet [16] represents a CNN methodology capable of simultaneously extracting FV features in both spatial and frequency domains. FVFSNet introduces a spatial-frequency information coupling module, strategically integrating the two domains to obtain high-quality fused features, thereby achieving significant advantages in accuracy.

Beyond purely CNN-based approaches, researchers have also explored the incorporation of other advanced network structures. Yang et al. [9] proposed FV-GAN, an FV identification method leveraging generative adversarial networks and eschewing fully connected layers in favor of a fully convolutional network. While this approach eliminates constraints on FV image size and reduces calculation time, the inherent challenges and instability in training generative adversarial networks persist. Additionally, to further improve the effectiveness of feature acquisition, Huang et al. [7] introduced Vision Transformer (ViT) [17] for the first time in the realm of FV identification, presenting a novel FVT model that effectively enhances identification accuracy. However, optimization challenges, particularly on small-scale datasets, are evident in the ViT model [18]. Notably, in scenarios where copious data and suitable pre-training models are lacking, the ViT architecture significantly lags behind the CNN architecture in performance.

Diverging from previous deep learning approaches, we integrate attention mechanisms into the design of the CNN, imparting it the capability to capture long-range feature dependencies within images. By adopting a large convolutional kernel structure LK Block, Let-Net efficiently captures the global features of finger veins more effectively, complementing the intrinsic ability of CNNs to express intricate details of local features. This successful integration of both local and global information significantly enhances the extraction and comprehension of complex FV features.

### 2.2. Kernel Size in Convolutional Layers

Following the advent of AlexNet, the integration of large kernels into CNN models has been infrequent. The preference for small convolutional kernels, characterized by their limited parameters and reduced computational cost, has swiftly positioned them as the primary choice for mainstream models. Contrary to this trend, recent research underscores the exceptional capabilities of large kernels across diverse vision tasks [19]. ERF (effective receptive field) theory [18] highlighted that larger kernels facilitate a broader receptive field, enabling the extraction of a more extensive range of information from input images. Empirical evidence from LRNet [20] substantiated that the incremental enlargement of kernel size correlates with gradual improvements in network performance, reaching optimal levels with larger kernels. GCN [21] employed a fusion of two convolutional methods to establish dense connections within expansive areas of the feature map, thereby further amplifying the kernel size in segmentation tasks. The FlexConv approach [22] extended kernel size by dynamically learning it during training and expediting large kernel convolutional operations using the Fourier transform. Liu et al. [23] drew inspiration from the Transformer design paradigm and introduced the ConvNets model, which systematically increases kernel size to elevate the depthwise convolutional layer, consequently enhancing performance. Despite the success of these models in high-level vision tasks, a notable experimental observation is their limited direct applicability to FV identification tasks. Consequently, we delve into the exploration of large kernel design in the context of FV identification. The objective is to extract more precise vein patterns, thereby augmenting identification performance.

### 2.3. Attention Mechanism

The attention mechanism, inspired by the human visual and cognitive system, finds applications in natural language processing, particularly for handling sequence data such as text, speech, and image sequences. In the realm of deep learning, incorporating the attention mechanism enables neural networks to autonomously learn and selectively emphasize crucial information within input data. This enhancement contributes to improved model performance and generalization capabilities. Attention mechanisms manifest in three primary types: self-attention, spatial attention, and channel attention. In computer vision, the channel attention mechanism stands out and has demonstrated noteworthy efficacy. For instance, Mnih et al. [24] innovatively integrated a deep neural network with an attention mechanism, introducing the RAM model. RAM utilizes RNN (recurrent neural network) for visual attention, predicting salient regions and iteratively updating the entire network in an end-to-end fashion through policy gradient. Some approaches amalgamate channel attention with spatial attention to yield favorable outcomes. Woo et al. [25] introduced the CBAM module, which can be seamlessly integrated with any CNN architecture. This module concatenates the channel attention sub-module and the spatial attention sub-module serially, with minimal additional computational cost. Recent studies affirm the attention mechanism’s efficacy in enhancing deep CNN. The synergistic integration of the attention mechanism with CNN architecture has proven advantageous across various visual tasks such as classification, detection, and segmentation. Notably, De et al. [26] amalgamated CNN and ViT to enhance accuracy, striving to establish an optimal plant disease detection model achieving high accuracy with reduced model size, without necessitating pre-training. This study adeptly combines convolutional blocks with attention mechanisms to integrate local and global information, facilitating more precise FV identification.

## 3. Proposed Method

This chapter provides a comprehensive exposition of Let-Net. Section 3.1 delineates the intricacies of the FV identification process, alongside an elucidation of the overarching network structure. Subsequently, Section 3.2 expounds upon the dual-channel network architecture, offering insights into its design and functionality. Section 3.3 delves into the particulars of the LK Block, shedding light on its structural components and operational characteristics. Finally, Section 3.4 elucidates the attention module employed within the Let-Net model, outlining its role and intricacies in the broader context of the network’s architecture.

### 3.1. Method Flow and Overall Network Structure

**Figure 1:**
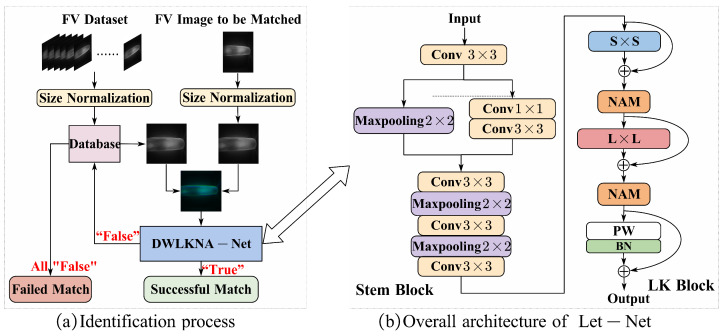
Method flow and overall structure of Let-Net: (**a**) Identification process; (**b**) Overall architecture of Let-Net.

In practical applications, the identification process of Let-Net is shown in Figure 1a, which mainly includes image input, image preprocessing, FV feature extraction, model prediction, and result matching. Initially, a representative sample image for each finger type is randomly selected from existing databases and incorporated into the new FV database, ensuring the inclusion of at least one sample image for each finger type. Subsequently, Let-Net receives the image to be detected and a specific type of image from the database. The model then generates a matching result, indicating the concordance or disparity between the two images. A “True” match signifies that the image to be detected and the selected database image originate from the same finger. Conversely, in the case of a “False” match, the next type of image is selected for further matching, iteratively continuing until the matching process is completed. If, after traversing the entire database, no matching image is found, it implies that the image to be detected fails to correspond to any type of image in the database. Through this approach, Let-Net efficiently leverages the image information within the database, progressively determining the category to which the detected image belongs, thereby accomplishing the FV identification task. In comparison to conventional classification models, Let-Net exhibits significant superiority in terms of scalability. When confronted with new finger samples not present in the training set, there is no need to retrain the model; rather, the integration of the vein feature data of these new fingers into the database enables successful and precise classification recognition for the respective fingers. In contrast, classification models typically undergo the process of data updating from the training set and model retraining when handling such new samples.

The network architecture of Let-Net, illustrated in Figure 1b, comprises two primary modules: the Stem Block and the Large Kernel Block (LK Block). Given an input FV image size of H×W×2, the image undergoes initial processing through the Stem Block, characterized by multiple convolutional layers and max-pooling layers. Following the 3×3 convolution, the feature map size becomes H×W×16. Subsequently, the feature map bifurcates into two paths: the first path undergoes max-pooling, while the second path undergoes 1×1 convolution and 3×3 convolution. At this juncture, the feature map size is $\frac{H}{2}\times \frac{W}{2}\times 16$, and the two resultant feature maps are concatenated. After traversing three convolutional layers and two max-pooling layers, the final output feature map size is $\frac{H}{8}\times \frac{Q}{8}\times 128$. The Stem Block facilitates downsampling, reducing dimensionality and compressing feature map information to enhance network efficiency.

After the Stem Block, the input progresses to the LK Block, where *L* and *S* denote the kernel sizes of the large convolution and auxiliary depthwise convolution, respectively. The kernel size transitions from the auxiliary kernel *S* to the large kernel *L*, employing pointwise convolution. The large kernel structure affords an ample effective receptive field and spatial aggregation capabilities, proving particularly adept at processing FV images with continuous texture information.

Furthermore, a NAM (neighborhood attention module) attention module is incorporated after each convolutional layer to channel the network’s attention towards channel and spatial locations rich in information content. Importantly, the LK Block maintains the feature map size, resulting in a final output feature map size of $\frac{W}{8}\times \frac{H}{8}\times 128$. Ultimately, the feature map undergoes expansion and traverses a fully connected layer, culminating in the output of two neurons representing the probabilities of “True” and “False”.

### 3.2. Dual-Channel Network Architecture

CNNs, represented by prominent architectures like VGG-Net, Google-Net, and ResNet, conventionally serve in the domain of classification tasks. Traditionally, these deep networks treat each image within a dataset as an independent sample, extracting salient features directly from individual images through non-linear transformations. In a departure from conventional methodologies, this research adopts an innovative strategy, incorporating a dual-channel network architecture. This unconventional choice stems from the aspiration to optimize the efficacy of constrained data resources and augment performance by expanding the pool of samples available for training purposes.

In the context of a dual-channel architecture, the input to the neural network is conceptualized as a pair of image patches. Consequently, each pair of images in the training set is amalgamated into a single training sample. This strategic approach results in an expansion of the number of training samples from *n* to the maximum $A_{n}^{2}$ (*n* represents the number of images in the training set, and the combination of two images ‘A’ and ‘B’ into sample pairs can occur in different orders, such as ‘AB’ and ‘BA’ representing distinct sample pairs). Implementing this network paradigm involves jointly feeding two images—originating from either the same or different fingers—into the network. The associated labels “True” or “False” signify whether each pair of images corresponds to the same or different fingers. During the testing phase, the initial input image serves as the template, while the second image functions as the test sample, yielding an output result of “True” or “False”.

The functionality of the dual-channel network architecture, akin to similarity measurement networks, is aimed at determining the similarity between two images. The distinction lies in our direct output of similarity results, whereas other similarity measurement networks, such as Triplet networks [27], extract FV features, followed by the calculation of metrics to decide whether two images belong to the same class. In constructing our network, we aim for a deeper integration of the processes of feature extraction and feature recognition, making it an implicit component within the network. The advantages of this approach manifest in two main aspects: On one hand, it simplifies the design requirements of the loss function, eliminating the need for excessive reliance on complex and explicit loss function designs. This not only reduces the design complexity but may also enhance the practical performance of the model, allowing the network to autonomously adjust feature representation and similarity measurement criteria from a global optimum perspective. On the other hand, it eliminates the need, in the post-training phase, for additional distance calculation steps or external classifiers such as support vector machines (SVM). This omission contributes to an overall improvement in the system’s efficiency and performance.

### 3.3. Design of the LK Block

Within CNNs, under equivalent depth conditions, large kernels can capture a broader range of spatial contextual information. This attribute is particularly crucial for biometric identification, such as FV identification, characterized by distinct textures and structural features. Through a larger convolutional operation, the model can more effectively capture locally significant features of larger dimensions within finger veins. This facilitates enhanced preservation of intricate details from the original image, providing a better reflection of the structural characteristics inherent in finger veins. Consequently, this contributes to an improved feature representation and ultimately enhances identification accuracy. However, the direct adoption of large kernels poses several challenges in practical applications. Firstly, the escalation of kernel size from 3×3 to 13×13 results in a substantial expansion of the model size, escalating computational overhead exponentially by 19 times. Secondly, as elucidated in [18,20], even meticulously designed large-kernel networks, when trained on extensive datasets, necessitate extensive optimization efforts with the inherent risk of performance degradation. This challenge becomes particularly pronounced in the context of FV network models, given the inherent constraints on training data size. For instance, the VERA dataset [13] comprises a mere 440 training samples. Thus, the direct application of large kernels proves impractical owing to limitations in computing resources and the intricate nature of model optimization. This predicament is especially formidable when addressing FV models characterized by constrained training data size.

Inspired by the concept of depthwise separable convolution, we introduce an LK Block that integrates three core components: hybrid depthwise convolution, residual connections, and pointwise convolution. The hybrid depthwise convolution comprises two depthwise convolutions—a primary large kernel with a size of L×L and an ancillary small kernel with a size of S×S. The incorporation of an auxiliary small kernel aims to capture fine-scale features within FV images, and the synergistic integration of both small and large kernels facilitates the amalgamation of features across different scales. For an input image with dimensions of $H\times W\times C_{in}$, the computational cost of this hybrid depthwise convolution is $H\times W\times C_{in}\times \left(L^{2}+S^{2}\right)\times 1$. Residual connections are employed to synergize large and small kernels, concurrently linking depthwise convolution and 1×1 pointwise convolution. Following hybrid depthwise convolution, pointwise convolution is introduced to facilitate the information flows across channels. Notably, pointwise convolution maintains the input dimension, with a computational cost of $H\times W\times C_{in}\times C_{in}$.

**Figure 2:**
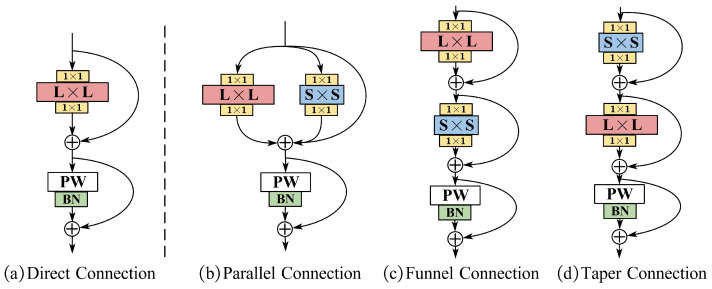
Structural design of large kernel: (**a**) Direct Connection; (**b**) Funnel Connection; (**c**) Funnel Connection; (**d**) Taper Connection.

To explore the most suitable large kernel feature extraction mode for finger veins, we introduce three distinct architectures for the LK Block, as illustrated in Figure 2. The ultimate Let-Net adopts the configuration depicted in Figure 2d. In this representation, *L* and *S* denote the kernel sizes for large convolution and auxiliary small depthwise convolution, respectively, while PW signifies pointwise convolution. The designs in columns b–d stem from the direct connections elucidated in column a. (1) Parallel connections (column b): Layer blocks incorporate parallel small cores within large depthwise convolutional layers. Pointwise convolutions are subsequently followed by parallel VGG-style convolutions, amalgamated with skip connections. (2) Funnel connection (column c): This configuration mirrors a ResNet-style layer block where the kernel size progressively diminishes from the large kernel *L* to the auxiliary kernel *S*. (3) Taper connection (column d): Resembling the funnel connection, this design applies hybrid convolutions in reverse order. Experimental results presented in Section 4.3.2 demonstrate that these three large kernel designs surpass conventional convolutional layers with ordinary small kernels in terms of performance, with only a negligible increase in computational effort. Notably, among these architectures, the taper connection exhibits superior performance and is consequently adopted as the final design:

(1) $$o_{1}=\sigma \left(x+Conv_{S\times S}^{dw}\left(x\right)\right)$$

(2) $$o_{2}=\sigma \left(o_{1}+Conv_{L\times L}^{dw}\left(o_{1}\right)\right)$$

(3) $$o=o_{2}+\sigma \left(Conv_{1\times 1}^{pw}\left(o_{2}\right)\right)$$

where *x* and *o* denote the input and output feature maps, respectively; $Conv_{L\times L}^{dw}$ and $Conv_{S\times S}^{dw}$ denote the depthwise convolution of the large kernel and small kernel, respectively; $Conv_{1\times 1}^{pw}$ denotes pointwise convolution; and σ denotes the activation function.

To align dimensions and introduce increased non-linear transformations, two sequences of 1×1 convolution operations are conducted within each LK Block. In each set of convolutional layers, the initial operation modifies the dimension of the input feature map from $C_{in}$ to $\alpha C_{in}$, followed by a subsequent transformation to $C^{\prime }$. This process incurs a computational cost of $H\times W\times C_{in}\times \alpha C_{in}+H\times W\times \alpha C_{in}\times C^{\prime }$. In the first set of convolutional operations, $C^{\prime }$ is equivalent to $C_{in}$, while in the second set, $C^{\prime }$ is equivalent to $C_{out}$. The cumulative computational cost for both sets of convolutions is expressed as $H\times W\times \alpha C_{in}\times \left(3C_{in}+C_{out}\right)$. Consequently, the total computational cost for an LK Block is delineated by Equation (4). To assess the computational efficiency, the computational cost ratio between the LK Block and a standard convolutional layer is calculated, as presented in Equation (5).

(4) $$H\times W\times \alpha C_{in}\times \left(L^{2}+S^{2}+C_{in}+\alpha \left(3C_{in}+C_{out}\right)\right)$$

(5) $$\begin{array}{cc} \frac{Cost_{ours}}{Cost_{normal}} & =\frac{H\times W\times \alpha C_{in}\times \left(L^{2}+S^{2}+C_{in}+\alpha \left(3C_{in}+C_{out}\right)\right)}{H\times W\times \alpha C_{in}\times C_{out}\times L^{2}}\\ \; & =\frac{1}{C_{out}}+\frac{1}{C_{out}}\frac{S^{2}}{L^{2}}+\frac{1+3\alpha }{L^{2}}\frac{C_{in}}{C_{out}}+\frac{\alpha }{L^{2}} \end{array}$$

In Equation (5), α, $C_{in}$, and $C_{out}$ are constants, and $C_{out}>>1$. In contrast to conventional large kernel structures, the design in this paper exhibits a significant computational cost advantage, with a complexity of only $O\left(\frac{1}{L^{2}}\right)$.

### 3.4. Attention Module

In FV images, the morphological attributes of veins frequently demonstrate a certain level of coherence. Leveraging attention mechanisms proves beneficial in capturing these long-range correlations, guiding the model to discern essential features finely while mitigating the impact of irrelevant ones. This aids the model in directing its attention toward key vein structures and detailed features, such as vein branches, orientations, and intersection points. Consequently, this approach contributes to enhancing the discrimination and robustness of features. To achieve this, the proposed Let-Net model astutely incorporates the attention mechanism, leveraging it to discover and reinforce dependencies between features at different locations within the image. Let-Net employs the normalized attention module (NAM) [28], an attention mechanism that assesses the importance of each feature dimension based on a batch-normalized scaling factor. Larger variances signify heightened dimensional variability and encapsulate richer information, thereby warranting greater attention. The introduction of this mechanism aids in optimizing feature selection, enhancing information flow within channel and spatial dimensions, and improving the model’s ability to integrate a wide range of features. Through this strategic approach, Let-Net can more attentively and accurately capture the crucial features of finger veins and reduce the interference of noises, thereby enhancing overall feature extraction performance. The standardized calculation and dimension weight formulas are articulated as follows:

(6) $$B_{out}=\gamma \frac{B_{in}-\mu_{B}}{\sqrt{\sigma_{B}^{2}+\varepsilon }}+\beta$$

(7) $$W_{c}=\frac{c_{i}}{\sum_{j=0}^{L}c_{j}}$$

(8) $$W_{s}=\frac{s_{i}}{\sum_{j=0}^{L}s_{j}}$$

**Figure 3:**
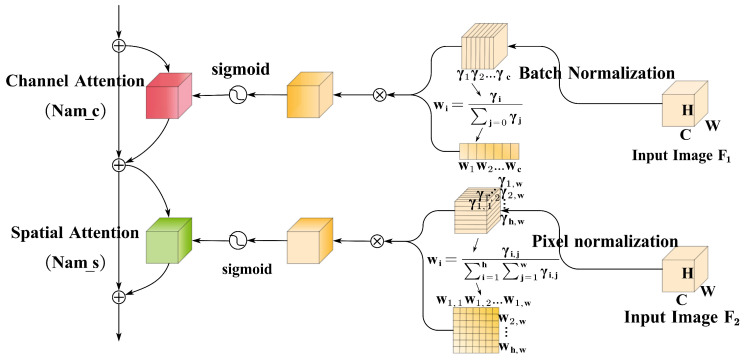
Channel attention mechanism and spatial attention mechanism.

In Equation (6), the variables $B_{in}$ and $B_{out}$ denote the input and output of the BN, respectively, while γ and β represent the scale and displacement. Additionally, $\mu_{B}$ and $\sigma_{B}$ signify the mean and standard deviation of the input data. In Equation (7), *i* represents the dimension, $c_{i}$ denotes the scaling factor of the dimension, *L* indicates the total length of the dimension, and $W_{c}$ represents the weight associated with the channel corresponding to dimension *i*. The application of the scaling factor from BN to the spatial dimension results in the derivation of the corresponding weight $W_{c}$, as illustrated in Equation (8). Typically, the dimensions calculated by BN align with channel dimensions. By determining the proportion of the scaling factor for each channel and multiplying it with the original features, the weight for each channel is computed, facilitating the redistribution of channel information. Ultimately, the channel attention is acquired by activating the sigmoid function, followed by dimension transformation to map the dimensions calculated by BN to spatial pixels. The channel attention mechanism based on normalization (NAM_c) and the spatial attention mechanism (NAM_s) are visually depicted in Figure 3.

## 4. Experiment and Result Analysis

### 4.1. Dataset Description

We use nine public datasets for evaluation experiments: SDUMLA [29], FV_USM [30], HKPU_FID [31], SCUT_RIFV [14], PLUSVein [32], MMCBNU_6000 [33], UTFVP [34], VERA [13], and THU_FVFVD [35].

(1) SDUMLA: This dataset contains images of 636 fingers. Each finger is captured six times, resulting in a total of 3816 FV images. The input of the dual channels is two pictures. Two pictures belonging to the same category are combined as positive samples, and two pictures of different categories are combined as negative samples. Without considering the order of channels, a total of 9540 positive samples can be formed, and a total of 7,269,480 negative samples can be formed. Considering that the number of negative samples is much larger than the number of positive samples, 9540 negative samples were randomly downsampled. The same applies to the following datasets.
(2) FV_USM: This dataset contains images of 492 fingers. Each finger was captured six times in a session, resulting in a total of 2952 FV images. (This dataset involves two stages, and only data from stage 1 is used for the experiments.)
(3) HKPU_FID: This dataset contains images of 312 fingers. Each finger is captured six times, resulting in a total of 1872 FV images.
(4) SCUT_RIFV: This dataset contains 606 images of fingers. Each finger is captured six times, resulting in a total of 3636 FV images. (This dataset involves three rolling poses and six illumination intensities, and only the subset under level 3 illumination with normal finger poses is used for the experiments.)
(5) PLUSVein: This dataset contains images of 360 fingers, with each finger captured five times, resulting in a total of 1800 FV images.
(6) MMCBNU_6000: This dataset contains images of 600 fingers. Each finger is captured ten times, resulting in a total of 6000 FV images.
(7) UTFVP: This dataset contains images of 360 fingers. Each finger is captured four times, resulting in a total of 1440 FV images.
(8) VERA: This dataset contains images of 220 fingers. Each finger is captured twice, resulting in a total of 440 FV images. Since the dataset is too small, we expanded it and randomly rotated and stretched each FV image to expand each finger image to 6, for a total of 1320 FV images.
(9) THU_FVD: This dataset contains 610 finger images. Each finger is captured eight times, resulting in a total of 4880 FV images. (This dataset involves two stages, and only data from stage 1 is used for the experiments.)

**Figure 4:**
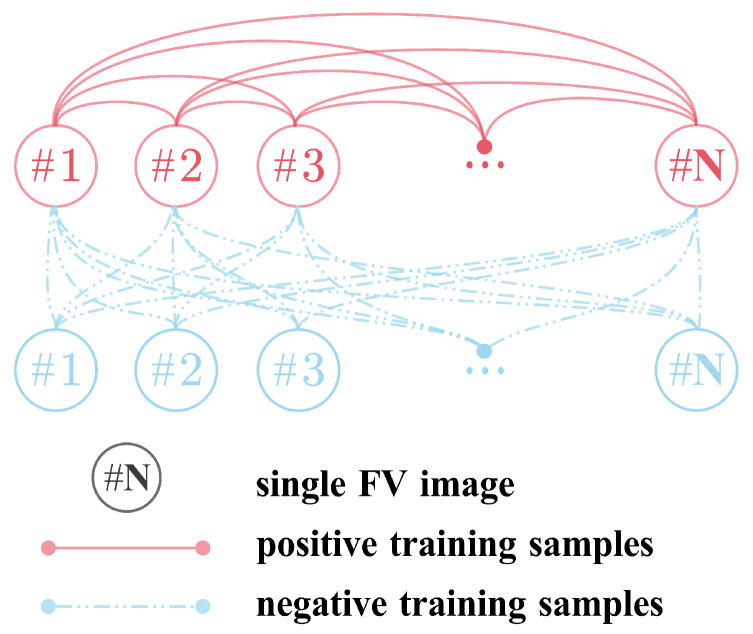
The way images are combined, where 1,2,3,…, N is the sequence number of a single finger vein image, and N is the number of a single finger vein image.

In this study, a dual-channel network is utilized, wherein each input consists of a pair of images, thereby yielding two images per pair. As illustrated in Figure 4, positive samples are constituted by pairing two images belonging to the same category, while negative samples are formed by combining images from different categories. Using the SDUMLA dataset as an example, a total of 19,080 positive samples and 14,538,960 negative samples can be generated. Given the substantial disparity in the number of negative and positive samples, a random undersampling is applied to the negative samples, reducing them to 19,080. The same procedure is followed for generating image pairs from eight additional datasets.

For the purpose of evaluation, a random split of the datasets into training and testing sets is executed using a 7:3 ratio. Furthermore, to ensure uniformity of features and maximize model performance, the input image dimensions for all datasets are normalized to 128×128 pixels.

### 4.2. Experimental Settings and Experimental Indicators

The utilized loss function during training is the binary cross-entropy loss, a widely adopted metric in the fields of machine learning and deep learning, particularly well-suited for binary classification problems. By minimizing the binary cross-entropy loss, the objective is to encourage the model to learn higher-quality decision boundaries for a more accurate differentiation of samples from different categories. For Let-Net, the minimization of binary cross-entropy loss drives the model to excavate more discriminative and abstract features, enabling it to comprehend the essential differences between two images and delineate decision boundaries for their differentiation more effectively.

The primary metric employed in the experimentation is the equal error rate (EER), a pivotal measure in biometric systems. EER is determined when the false acceptance rate (FAR) equals the false rejection rate (FRR). Falsely accepted pairs arise when two FV images, belonging to different categories, are erroneously identified as being in the same category. FAR quantifies the percentage of falsely accepted pairs relative to all inter-class pairs, essentially representing the proportion of “unmatched FV images treated as matching FV images”. Conversely, falsely rejected pairs occur when two FV images from the same class are erroneously identified as belonging to different classes. FRR is the percentage of falsely rejected pairs among all within-class pairs, signifying the instances where “FV images that should be matched are not considered matched FV images”. The computations for FAR and FRR are articulated in Equations (9) and (10), respectively.

(9) $$FAR=\frac{N_{FA}}{N_{IRA}}\times 100\%$$

(10) $$FRR=\frac{N_{FR}}{N_{GRA}}\times 100\%$$

where $N_{FA}$ and $N_{FR}$ are the number of false acceptances and false rejections, and $N_{IRA}$ and $N_{GRA}$ are the total number of inter-class tests and intra-class tests. In addition to EER, we employ the accuracy rate (ACC) as an additional evaluative criterion.

The deep learning model employed in the experimental setup is implemented through the TensorFlow framework. The computational infrastructure comprises an RTX2080ti GPU, and the operating system utilized is Ubuntu 18.04. To mitigate overfitting and enhance the network’s learning and detection capabilities, a pre-trained model initializes the Stem Block, and the training batch size is set to 32. For the initialization of convolutional kernel and depthwise convolutional kernel parameters, the standardized Glorot initialization method is employed. The parameters for the Adam optimizer are configured as follows: a learning rate of 0.0001; an exponential decay rate for the first-order moment estimation (beta1) of 0.9; an exponential decay rate for the second-order moment estimation (beta2) of 0.999; and epsilon set to $1\times 10^{-7}$, where this parameter serves the purpose of preventing division by zero.

### 4.3. Results Evaluation and Comparison

This section undertakes a thorough performance evaluation of Let-Net, conducting experiments across nine publicly available datasets. The evaluation involves both quantitative and qualitative comparisons with other existing methods.

#### 4.3.1. Comparison and Evaluation with Existing FV models

**Figure 5:**
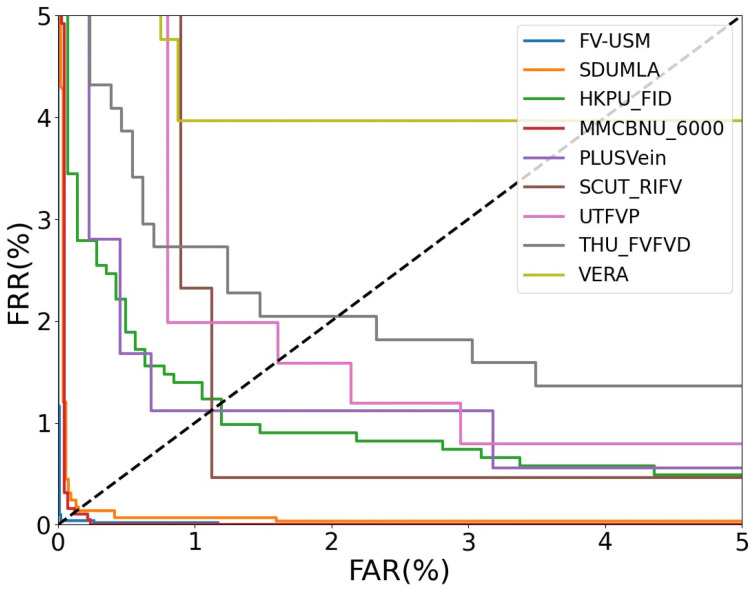
ROC curves of Let-Net on nine FV datasets.

To assess the efficacy of Let-Net, we conducted a comparative analysis with the SOTA deep-learning-based FV identification models. The benchmark models include FV_CNN [8], a reference to a CNN architecture designed for vein identification; Fvras-net [9], an embedded FV identification system; FV code [36], a method employing FV code indexing; L-CNN [37], a lightweight CNN model; ArcVein [38], which introduces a novel loss function, Arcvein loss; FVSR-Net [39], a model integrating a bio-optical model with a multi-scale CNN E-Net; S-CNN [34], a novel shallow CNN model; FVT [7], a transformer-based deep model with pioneering experiments across nine datasets; L-S-CNN [40], a lightweight Siamese network with self-attention mechanism; and FVFSNet [16], a method that concurrently extracts FV features in the spatial and frequency dimensions. Employing EER as a metric, comparative experiments were conducted across the nine public FV datasets outlined in Section 4.1, with the results presented in Table 1. The receiver operating characteristic curves (ROC) of the proposed Let-Net on the nine FV datasets are shown in Figure 5. The outcomes reveal significant advantages of Let-Net when compared with several advanced models. Notably, on the FV_USM and SDUMLA datasets, Let-Net reduces the EER by 0.91% and 1.56%, respectively, in comparison to the Fvras-net algorithm [9]. Experimental findings demonstrate that Let-Net exhibits a lower EER compared to the majority of FV identification methods, providing evidence for the effectiveness and generalization capability of Let-Net. Furthermore, in comparison to the SOTA FVFSNet [16], Let-Net demonstrates competitive results, achieving optimal performance on seven datasets, excluding SCUT_RIFV and HKPU_FID. The approach employed by FVFSNet, which achieves outstanding accuracy by integrating spatial and frequency domain features while maintaining a lightweight architecture, is noteworthy. This innovative perspective can also be applied to similarity network architectures, presenting a promising direction for FV identification. Let-Net, on the other hand, focuses its attention on spatial domain features, achieving remarkable results by combining global and local information to filter more high-quality features. Furthermore, the FVFSNet model exhibits a total parameter count of 1.4 M and a FLOPs of 623 M. In comparison, Let-Net’s total parameter count is approximately two-thirds that of FVFSNet, with a relatively lower FLOPs value. This implies that Let-Net not only maintains advantages in terms of model size, demonstrating lightweight characteristics, but also possesses lower computational complexity, making it more suitable for deployment in resource-constrained environments.

#### 4.3.2. Ablation Experiments

To elucidate the significance of each constituent in the network model design, we conducted three sets of ablation experiments, the outcomes of which are presented in Table 2. The designations highlighted in bold fonts in the table denote components incorporated into the final model.

**Kernel Size:** The initial exploration assesses the impact of diverse kernel sizes on identification performance. Through manipulating the size of the large kernel, the experiment discerned optimal results when the large kernel size was set to 13×13. Despite the intuitive concern that overly large kernel sizes may adversely affect feature extraction given a feature map size of 16×16×128, practical findings demonstrated that large-sized kernels can adapt to small feature maps and even enhance results. For the FV identification task, a larger kernel size minimally impacts experimental outcomes and even a size of 31×31 yields satisfactory results. However, considering accuracy and computational cost, a kernel size of 13×13 is ultimately selected.

**Components of Let-Net: **Table 2 clearly shows the impact of the three components of Stem Block, LK Block, and NAM on recognition performance. The absence of LK Block leads to a 3.12% reduction in accuracy for the SDUMLA dataset. This is attributed to the susceptibility of FV images to factors such as lighting variations and noise. The utilization of large kernels during the feature extraction phase facilitates the integration of a broader range of neighborhood information, enabling the model to effectively adapt to these variations. This, in turn, enhances the robustness and stability of the recognition system. Moreover, large kernels excel in preserving fine-grained details from the original images, particularly in the context of processing texture-rich FV images. This capability contributes to an improved feature representation, consequently elevating the overall recognition accuracy. The adoption of the LK Block thus proves advantageous in addressing the challenges posed by illumination and noise in FV image processing. The attention module NAM exerts a profound impact on FV identification, as the removal of NAM results in a direct accuracy drop to 95.17% for SDUMLA. Upon analysis, the incorporation of NAM facilitates a more focused attention of the model on vital vein structures and intricate features, consequently elevating both the discriminative capacity and robustness of the extracted features. While the Stem Block minimally affects experimental results, it demonstrates a certain degree of generalization effect. Let-Net synergistically leverages the advantages of the Stem Block, LK Block, and NAM. The integration of large kernels and attention mechanisms enables Let-Net to effectively learn and extract FV features, achieving commendable results on both datasets with a high identification rate.

**Architecture of the LK Block:** The investigation into three hybrid architecture designs (Section 3.3) is detailed in Table 2. The taper connection design outperforms others, showcasing the potent ability of large kernels to optimize FV identification networks. In contrast to some existing work, as evidenced in the study [19], which underscores the efficacy of the parallel reparameterized structure in high-level tasks like image classification, this study finds that the taper connection design yields superior performance. Two potential reasons account for this disparity. Firstly, there are differences in dataset distribution and quantity, where datasets like ImageNet possess vast amounts of data, while FV datasets comprise significantly fewer samples, posing challenges in optimizing large kernels. Secondly, the task focus diverges, with high-level visual tasks emphasizing semantic information over pixel correspondence between images, as compared to the FV identification task.

#### 4.3.3. Comparative Experimental Results between Let-Net and Classic Models

**Figure 6:**
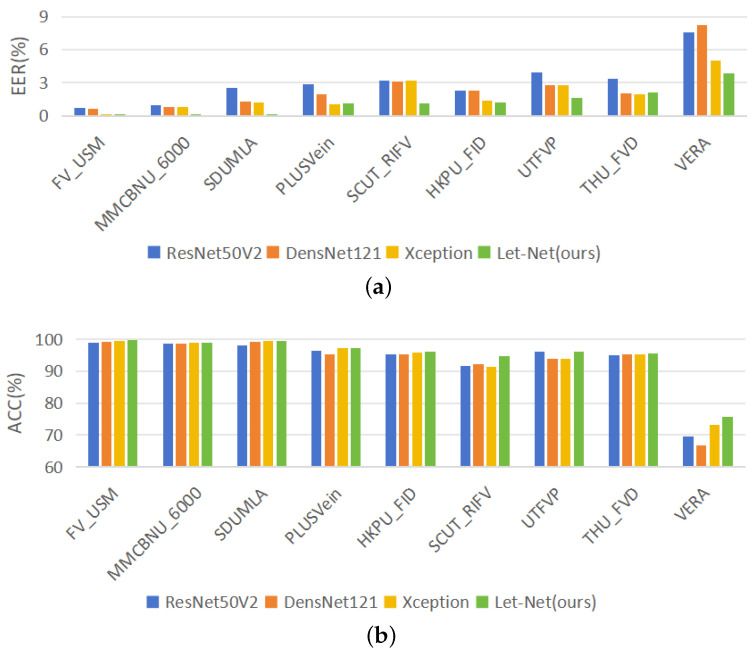
Bar charts of the results of classical models on nine FV datasets: (**a**) bar charts of the EERs, (**b**) bar charts of the ACC.

This section employs two evaluation metrics, EER and ACC, to conduct verification experiments across nine publicly accessible FV datasets. The results are comprehensively presented in Table 3. Figure 6a,b illustrate the performance comparison between Let-Net and classical models in terms of EER and ACC, respectively, through bar charts. To establish an intuitive and comparable benchmark, a selection of other deep convolutional network models with analogous network structures is chosen for comparison, including the well-established ResNet50V2, DensNet121, and Xception. The rationale behind choosing these models for comparison lies in their proximity to Let-Net in terms of parameter count and computational cost. Experimental findings reveal that Let-Net consistently outperforms other models in terms of ACC and EER across multiple datasets. Notably, on the MMCBNU_6000 and SCUT_RIFV datasets, Let-Net achieves an EER of only 0.12% and 1.12%, respectively, markedly lower than its counterparts, signifying its robust capability in minimizing EER. Particularly noteworthy are the ACC scores on the SDUMLA and FV_USM datasets, where Let-Net attains ACC values of 99.5% and 99.77%, respectively—remarkable improvements in comparison to other models.

In practical deployment scenarios, particularly in light of the widespread utilization of edge computing devices characterized by constrained resources as the target platform, significant disparities commonly arise between the hardware configuration of these devices and the research and development environment utilized for model training and optimization. For a comprehensive evaluation of the efficacy and applicability of the Let-Net model in real-world settings, this study systematically quantifies key metrics, specifically the parameter count (Params) and the floating point operations (FLOPs). As illustrated in Table 4, Let-Net demonstrates noteworthy lightweight characteristics when juxtaposed with several classical models. Boasting a mere 0.89M parameters, it attains a controlled total of 0.25G floating point operations, positioning itself as one of the most resource-efficient models.

#### 4.3.4. Computational Cost

This section conducts experiments to assess the processing time of various datasets using the dual-channel architecture of classical networks. Utilizing the SDUMLA and FV_SUM datasets as exemplars, the time expended in each training round for prominent models, including VGG, ResNet, and Xception, was computed. The outcomes are presented in Table 5 and Table 6, where “Training” and “Prediction” indicate the time required for a training round and predicting the entire test set, respectively. “Total” represents the sum of training and prediction time, while “Single batch time” signifies the time spent training a single batch of samples. Across both the FV_USM and SDUMLA datasets, Let-Net exhibits lower time consumption compared to other deep learning models. This can be attributed to several factors. Firstly, the Stem Block employs depthwise convolution, traditional convolution, and max-pooling to reduce the size of the input feature map. Max-pooling downsamples the feature map, while convolution increases the dimension to prevent excessive loss of feature information. Moreover, the extensive use of depthwise convolution in the Stem Block significantly reduces the number of parameters compared to traditional convolution, enhancing the model’s generalization ability. Secondly, the LK Block’s core module employs the principles of deep convolution, and the large kernel itself minimally impacts the parameter count. Lastly, Let-Net’s reliance on a single fully connected layer with only two output neurons reduces parameters compared to mainstream network models like VGG and ResNet. While these models boast tens of millions of parameters, Let-Net maintains a lightweight profile with only tens to hundreds of thousands of parameters. Consequently, the method we proposed demonstrates lower computational cost and memory requirements than other network models.

## 5. Summary and Outlook

This study presents Let-Net, an FV identification model that combines both local and global information. By incorporating a dual-channel architecture to expand the dataset scale, Let-Net employs large kernels to capture a more extensive spatial context. Additionally, it integrates attention mechanisms to enhance information flow within the channel and spatial dimensions, aiming to extract precise vein features while maintaining a lightweight characteristic. Experimental results demonstrate Let-Net’s effectiveness in enhancing identification accuracy and reducing misidentification rates, surpassing current SOTA methods across multiple datasets and exhibiting robust generalization. Notably, on the SDUMLA and FV_USM datasets, Let-Net achieves identification accuracies of 99.5% and 99.77%, with EER of only 0.15% and 0.04%, ranking it first among published methods. Moreover, Let-Net demonstrates cost-effectiveness in terms of network parameter complexity, boasting parameter size and FLOPs of only 0.89M and 0.25G compared to other CNN-based models.

Let-Net, characterized by its lightweight and high-precision features, proves to be particularly suitable for deployment on edge devices or small terminals with limited memory and computational capabilities. Aligned with the current emphasis on data security and user privacy protection, Let-Net’s lightweight nature naturally aligns with the principles of federated learning (FL). FL aims to optimize models globally while preserving user privacy by training models on device edges and aggregating model updates on a central server without collecting raw data. Applying Let-Net within an FV framework not only mitigates security risks associated with data transmission and storage but also enhances model performance and user data security in distributed environments through efficient collaborative training. However, despite Let-Net’s lightweight characteristics, deployment on extremely resource-limited embedded devices or mobile platforms necessitates consideration of the computational resources (CPU/GPU/memory) required for model execution. Further optimization of model size or the introduction of knowledge distillation may be necessary in such scenarios.

## Figures and Tables

**Table 1:** Comparison with other FV models.

	EER (%)								
	FV_USM	SDUMLA	MMCBNU_6000	HKPU_FID	THU_FVD	SCUT_RIFV	UTFVP	PLUSVein	VERA
FV_CNN [8]	-	6.42	-	4.67	-	-	-	-	-
Fvras-net [9]	0.95	1.71	1.11	-	-	-	-	-	-
FV code [36]	-	-	-	3.33	-	-	-	-	-
L-CNN [37]	-	1.13	-	0.67	-	-	-	-	-
ArcVein [38]	0.25	1.53	-	1.30	-	-	-	-	-
FVSR-Net [39]	-	5.27	-	-	-	-	-	-	-
S-CNN [41]	-	2.29	0.47	-	-	-	-	-	-
FVT [6]	0.44	1.50	0.92	2.37	3.60	1.65	1.97	2.08	4.55
L-S-CNN [40]	0.19	0.59	0.12	-	-	-	-	-	-
FVFSNet [16]	0.20	1.10	0.18	**0.81**	2.15	0.83	2.08	1.32	6.82
Let-Net (ours)	**0.04**	**0.15**	**0.12**	1.54	**2.13**	**1.12**	**1.58**	**1.12**	**3.87**

Notes: Numbers in bold indicate the minimum EER.

**Table 2:** Ablation experiment.

	Method	FV_USM EER (%)	SDUMLA EER (%)	Parameters (M)
Kernel Size	7×7	99.57	99.10	0.72
	11×11	99.66	99.35	0.81
	** 13×13 **	**99.77**	**99.42**	**0.89**
	17×17	99.68	99.34	1.08
	31×31	99.66	99.33	1.67
Components of Let-Net	No Stem	98.25	97.86	0.51
	No LK	96.65	96.27	0.78
	No NAM	95.76	95.17	0.66
	No Stem or LK	94.71	94.16	0.52
	No Stem or NAM	93.64	93.11	0.27
	No LK or NAM	88.12	87.76	0.55
	**Stem, LK, and NAM**	**99.77**	**99.50**	**0.89**
Architecture of the LK Block	Direct Connection	96.32	96.01	0.88
	Parallel Connection	98.46	97.26	0.89
	Funnel Connection	98.26	97.49	0.89
	**Taper Connection**	**99.77**	**99.50**	**0.89**

Notes: Bold font indicates the optimal structure and its corresponding parameter values.

**Table 3:** Comparison with the classical models.

	ResNet50V2		DensNet121		Xception		Let-Net	
	EER (%)	ACC (%)	EER (%)	ACC (%)	EER (%)	ACC (%)	EER (%)	ACC (%)
MMCBNU_6000	0.97	98.63	0.76	98.60	0.82	**98.86**	**0.12**	98.84
HKPU_FV	2.26	95.24	2.27	95.23	1.36	95.74	**1.21**	**96.10**
VERA	7.56	69.66	8.23	66.75	5.05	73.11	**3.87**	**75.60**
UTFVP	3.91	96.24	2.81	93.74	2.80	93.80	**1.58**	**96.18**
THU_FVD	3.32	94.97	2.02	95.30	**1.99**	95.35	2.13	**95.52**
SCUT_RIFV	3.20	91.56	3.09	92.12	3.17	91.23	**1.12**	**94.69**
FV_USM	0.75	98.90	0.65	99.10	0.15	99.35	**0.04**	**99.77**
SDUMLA	2.52	98.08	1.31	99.13	1.18	99.36	**0.15**	**99.50**
PLUSVein	2.85	96.27	1.97	95.16	**1.01**	97.15	1.12	**97.32**
Average	3.04	93.28	2.57	92.79	1.95	93.77	**1.26**	**94.84**

Notes: Numbers in bold indicate the minimum EER.

**Table 4:** Comparison of Let-Net with the classical models regarding parameters and FLOPs.

Model	Params (M)	FLOPs (G)	EER (%) *	ACC (%) *
ResNet50V2	23.63	6.99	3.04	93.28
DensNet121	7.07	5.70	2.57	92.79
Xception	2.09	16.8	1.95	93.77
Let-Net (ours)	0.89	0.25	1.26	94.84

* The average values of nine FV datasets.

**Table 5:** Processing time on FV_USM.

	Training (s)	Prediction (s)	Total (s)	Single Batch Time (ms)
VGG16	10	5	15	48
VGG19	12	6	18	55
Resnet50V2	11	6	17	53
InceptionV3	16	9	25	78
DensNet121	22	11	33	102
Xception	19	6	25	77
RepLKNet	96	6	120	373
Let-Net (ours)	3	3	6	17

**Table 6:** Processing time on SDUMLA.

	Training (s)	Prediction (s)	Total (s)	Single Batch Time (ms)
VGG16	13	7	20	48
VGG19	15	8	23	55
Resnet50V2	14	9	23	54
InceptionV3	20	12	32	77
DensNet121	26	15	41	97
Xception	25	7	32	78
RepLKNet	126	32	158	379
Let-Net (ours)	4	3	7	18

## Data Availability

Data are contained within the article.
